# Traumatic brain injury and hyperglycemia

**DOI:** 10.18632/oncotarget.15740

**Published:** 2017-02-26

**Authors:** Daniel Vieira de Oliveira, Robson Luis Amorim, Rita de Cássia Almeida Vieira, Wellingson Silva Paiva

**Affiliations:** Department of Neurology, School of Medicine, University of São Paulo (USP-SP), São Paulo, Brazil

We read with great interest the recent study by Shi et al [1] published in the *Oncotarget*. The management of modifiable risk factors in traumatic brain injury victims has a main role in the patients outcome and a inappropriate treatment is followed by a worse prognosis. Knowledge about the mechanism of hyperglycemia, comorbidities, potential causes, effects and associated factors can improve the treatment process associated with adequate therapeutics could reduce the complications and modify outcome of these victims [1, 2].

A strong point of the paper is providing a meaningful view of mechanisms of hyperglycemia after traumatic brain injury and the pathway that lead to systemic dysfunctions and the role of comorbidities, including iatrogenic factors [1]. However, in spite of the paper covers some themes, a important consideration that should be mentioned in this review is the definition of papers selection criteria [3, 4].Hence, the use of a reproducible methodology in the construction of systematic review provides better scientific evidence and increase quality information [3, 4].Other important point of concern is the concepts covered in the study. In trauma, especially those of severity in traumatic brain injury, stratification methods for standardized clinical assistance and research are widely disseminated in the medical literature [2, 5].

Liu-DeRyke et al [6] described clinical impact of early hyperglycemia during acute phase of traumatic brain injury, probably result from action of high glucose toxicity on neurons. The authors also have discussed several diagnosis and mechanisms of hyperglycemia, there are references to controversy regarding the use of intensive insulin therapy [7], but the authors did not present the best management of hyperglycemia for clinical use, which would make the review more complete and useful. Identify and delimit what are the limits for hyperglycemia (range of serum glucose), the epidemiological and clinical characteristics of the population of these studies [4]. We believe that future studies are necessary to evaluate treatment protocols of serum glucose target levels. However, these do not exclude the relevance of the results of this interesting paper.

The review described by Shi et al [1] showed the importance of studying the hyperglycemia as a risk factor that could be modified by therapeutic measures. The correlation among the level of serum glucose, management of hyperglycemia and outcome remains on developement. This is a complex issue, presents particularities, and perhaps should be stratified according to level of blood glycemia, severity of TBI, length of hospitalization, outcome and comorbidities.
